# Age-related changes in patients with upper limb thalidomide embryopathy in the United Kingdom

**DOI:** 10.1177/17531934231164093

**Published:** 2023-04-06

**Authors:** Magdalena Markiewicz, Paul Stirling, Susan Brennan, Geoffrey Hooper, Wee Lam

**Affiliations:** 1 Edinburgh Medical School, The University of Edinburgh, Edinburgh, UK; 2 Fife Hand Clinic, Queen Margaret Hospital, Dunfermline, UK; 3 Thalidomide Trust, Saint Neots, UK; 4 Retired hand surgeon; 5 Royal Hospital for Children and Young People, Edinburgh, UK

**Keywords:** Thalidomide, thalidomide embryopathy, upper limb embryopathy, thalidomide patients

## Abstract

We report the  long-term upper limb disability, health-related quality of life (HRQoL), functional impairment, self-perception of appearance and prevalence of neuropathic pain in patients with upper limb thalidomide embryopathy in the United Kingdom. One-hundred and twenty-seven patients responded to our electronic questionnaire. Mean Quick Version of the Disabilities of Arm, Shoulder, and Hand score was 54.3 (SD 22.6). Median EuroQoL 5-Dimension 5-Likert index, Work and Social Adjustment Scale, Derriford Appearance Scale 24 and Neuropathic Pain Scale were 0.6 (IQR 0.4 to 0.7), 15.5 (IQR 8.0 to 23.5), 35.5 (IQR 28.0 to 50.5), and −0.8 (IQR −1.4 to 0.8), respectively. Thirty-three patients (26%) reported neuropathic pain. Finger changes associated with radial longitudinal deficiency were an independent predictor of more severe upper limb disability. Eighty-nine patients (70%) reported deteriorating HRQoL with increasing age. Patients with upper limb thalidomide embryopathy experience age-related worsening of symptoms and function, highlighting the need for ongoing specialist care and support.

**Level of evidence:** IV

## Introduction

Thalidomide was marketed worldwide as a non-addictive, non-toxic and non-barbiturate sedative in the 1950 s and 1960 s (Mansour et al., 2018; Vargesson, 2015). Routine drug screening of thalidomide in rodents demonstrated acceptable drug safety; however, no screening was undertaken in humans before approval by drug licencing authorities (Brent and Holmes, 1988; Vargesson, 2015). Thalidomide gained popularity as an effective drug for relieving morning sickness in expectant mothers (Mansour et al., 2018; Vargesson, 2015). In 1960, it was suggested that an epidemic of congenital upper limb differences was linked to maternal ingestion of thalidomide (Lenz and Knapp, 1962; McBride, 1961). The evidence continued to grow, and by late 1961 the teratogenic effects of thalidomide were indisputable (Vargesson, 2015). Maternal ingestion of thalidomide during the time-sensitive window of 20 to 36 days post-conception led to congenital abnormalities in multiple tissues and organs, collectively referred to as thalidomide embryopathy. The eyes, ears, genitals, vertebral column, internal organs, facial nerves and limbs were commonly affected (Newman, 1986; Vargesson, 2018). The most striking teratogenic effects of thalidomide were seen in the upper limb, with congenital upper limb differences occurring in up to 87% of patients with thalidomide embryopathy (Newman, 1986; Vargesson, 2018). Upper limb thalidomide embryopathy followed a predictable pattern, with the thumb being the most sensitive to thalidomide followed by the radius, humerus, ulna and fingers on the ulnar aspect of the hand (Newman, 1986; McCredie and Willert, 1999). The congenital upper limb differences seen therefore ranged from underdevelopment of the thumb (thumb hypoplasia) to complete absence of the upper limb (amelia). Upper limb thalidomide embryopathy was typically bilateral and more severe in the left than right upper limb; however, the reasons for this observation remain unknown (Newman, 1986; Vargesson, 2018).

Thalidomide was marketed in the United Kingdom (UK) between April 1958 and November 1961 (Mansour et al., 2018; Vargesson, 2018). Most children with thalidomide embryopathy were born between January 1959 and August 1962; however, some were born as late as May 1963 as thalidomide continued to be circulated in the community (Mansour et al., 2018). Over 2000 children with thalidomide embryopathy were born in the UK, and around half died within the first few months of life (Smithells and Newman, 1992). Now aged between 59 and 64 years, around 450 patients remain in the UK. Many patients with congenital upper limb differences were treated surgically as children; however, the long-term effects of upper limb thalidomide embryopathy remain unknown.

The primary aim of this study was to report the long-term upper limb disability in patients with upper limb thalidomide embryopathy in the UK. The secondary aims were to investigate the health-related quality of life (HRQoL); functional impairment in terms of work, home management, social leisure activities, private leisure activities and close relationships; self-perception of appearance; and prevalence of neuropathic pain within this group.

## Methods

### Participants

Participants were living patients with thalidomide embryopathy identified from records held by the Thalidomide Trust, a registered charity in the UK. The inclusion criterion was defined as the presence of congenital upper limb differences secondary to thalidomide exposure. Informed consent was obtained from all patients before participation in this study. Written consent was obtained from patients who provided written responses electronically and verbal consent was obtained from patients who provided verbal responses via telephone interviews. Patients received information about the purpose of the study, procedure, data handling and right to withdrawal. Patients received no payment or incentive for participation in this study. Ethical advice and permission were granted from the local ethics committee of the National Health Service (NHS) NHS Lothian, United Kingdom and Thalidomide Trust. As patients do not belong to one particular hospital, the study was conducted in accordance with the principles of the Declaration of Helsinki regarding consent and data protection.

### Data collection

An electronic questionnaire was developed with input from the Thalidomide Trust and a multidisciplinary team within NHS Lothian, UK consisting of hand surgeons, hand therapists, congenital hand nurse practitioners and occupational therapists. The questionnaire was piloted to four patients before starting data collection to ensure that the questions were relevant and the language of the questionnaire was appropriate.

The questionnaire was sent to 346 patients, identified by the Thalidomide Trust as patients with upper limb thalidomide embryopathy. Details of each patient were stored by the Thalidomide Trust; however, no demographic data was collected as complete anonymity was requested to be maintained throughout. However, it was known that the age range of patients was 59 to 64 years.

Data were collected over a 3-month period between July and October 2021. Responses were entered electronically into a secure electronic database. Patients who were unable to respond were offered a telephone call with an independent member of staff from the Thalidomide Trust to complete the questionnaire verbally.

The severity of upper limb thalidomide embryopathy affecting each patient was determined using lay descriptions that approximately corresponded to the Oberg–Manske–Tonkin (OMT) classification of congenital upper limb differences (Goldfarb et al., 2020). Substantial intra-rater and inter-rater reliability of the OMT classification has been demonstrated in a previous study (Sletten et al., 2022). Each patient selected applicable lay-term descriptions of their congenital upper limb differences and symptoms from a list included in the questionnaire.

### Outcome measures

In order to assess long-term upper limb disability and function, we collected a range of patient-reported outcome measures (PROMs). The primary outcome measure was the Quick Version of Disabilities of Arm, Shoulder, and Hand (QuickDASH) questionnaire. This PROM assesses upper limb disability and ranges from 0 to 100, where 0 indicates no disability and 100 indicates the most severe disability (Beaton et al., 2005). The secondary outcome measures were the EuroQoL 5-Dimension 5-Likert (EQ-5D-5L) index, Work and Social Adjustment Scale (WSAS), Derriford Appearance Scale 24 (DAS-24), and Neuropathic Pain Scale (NPS). The EQ-5D-5L index assesses HRQoL and ranges from −0.594 to 1, where scores below 0 indicate a health state ‘worse than death’ and scores of 1 indicate ‘perfect health’ (Rabin and Charro, 2001). The WSAS assesses functional impairment in terms of work, home management, social leisure activities, private leisure activities and close relationships. It ranges from 0 to 40, with higher scores indicating greater functional impairment (Mundt et al., 2002). The DAS-24 assesses self-perception of appearance and ranges from 11 to 96, with higher scores indicating poorer self-perception of appearance (Carr et al., 2005). The NPS assesses for the presence or absence of neuropathic pain, it generates a discriminant function score that ranges from −1.408 to 2.792. Scores below 0 indicate non-neuropathic pain and scores at or above 0 indicate neuropathic pain (Krause and Backonja, 2003). Data regarding previous surgical treatment, deterioration in HRQoL with increasing age and difficulty accessing healthcare services were also collected, as these have been shown to be relevant in this group in a previous study (Nippert et al., 2002). Deterioration in HRQoL with increasing age pertains to general self-perceived deterioration over time rather than over a categorized fixed time period.

### Statistical analysis

Data were tested for normality using the Shapiro–Wilk test. Continuous data are presented as means with the standard deviation (SD) where normally distributed and as medians with the interquartile range (IQR) where not normally distributed. Categorical data are summarized as frequencies and percentages.

The QuickDASH score, EQ-5D-5L index, WSAS, DAS-24 and NPS were calculated for each patient in accordance with published guidelines (Beaton et al., 2005; Carr et al., 2005; Krause and Backonja, 2003; Mundt et al., 2002; Rabin and Charro, 2001). It was not possible to calculate six QuickDASH scores, one EQ-5D-5L index, three WSAS, seven DAS-24 and 23 NPS as some patients provided incomplete responses. Missing data were accounted for using complete case analysis, meaning that patients were excluded if incomplete responses were provided for any of the outcome measures included in the analysis.

The impacts of each congenital upper limb difference, having multiple congenital upper limb differences, and receiving previous surgical treatment on the QuickDASH score, EQ-5D-5L index, WSAS, DAS-24 and NPS were investigated using multiple regression analysis. Univariate analysis was initially performed. The independent samples *t*-test and Mann–Whitney U-test were used for parametric and non-parametric data, respectively. Variables found to be statistically significant at the level *p *< 0.05 were included in the multiple regression analysis. The *beta* coefficients, 95% confidence intervals and *p*-values are presented for each variable. The relationship between the QuickDASH score and EQ-5D-5L index was investigated using Spearman’s correlation. A *p*-value of less than 0.05 was considered to represent statistical significance.

## Results

### Participant characteristics

A total of 127 patients responded to the questionnaire (37% response rate). One-hundred and twenty patients (95%) had bilateral congenital upper limb differences. The prevalence of different congenital upper limb difference patterns is presented in Table 1. Ninety-five patients (75%) had multiple congenital upper limb differences.

**Table 1.** Prevalence of different congenital upper limb difference patterns.

Congenital upper limb difference (OMT classification)	*n* (% of all patients)
Transverse deficiency (I-A-1-iii)	19 (15)
Amelia (I-A-1-iii-a)	17 (13)
Unilateral	4 (3)
Bilateral	13 (10)
Segmental (I-A-1-iii-b)	2 (2)
Unilateral	0 (0)
Bilateral	2 (2)
Intersegmental deficiency (I-A-1-iv)	98 (77)
Proximal (I-A-1-iv-a)	14 (11)
Unilateral	6 (5)
Bilateral	8 (6)
Distal (I-A-1-iv-b)	17 (13)
Unilateral	5 (4)
Bilateral	12 (9)
Proximal and distal (I-A-1-iv-c)	67 (53)
Unilateral	19 (15)
Bilateral	48 (38)
Radial longitudinal deficiency (I-A-2-i)	57 (45)
Unilateral	17 (13)
Bilateral	40 (31)
Thumb hypoplasia (I-B-2-i)	93 (73)
Unilateral	15 (12)
Bilateral	78 (61)
Associated with radial longitudinal deficiency	53 (42)
Finger changes	70 (55)
Unilateral	21 (17)
Bilateral	49 (39)
Associated with intersegmental deficiency	58 (46)
Associated with radial longitudinal deficiency	42 (33)
Associated with thumb hypoplasia	58 (46)

OMT classification: Oberg–Manske–Tonkin classification.

### QuickDASH score

The mean QuickDASH score was 54.3 (SD 22.6), indicating considerable upper limb disability. Univariate analysis confirmed that radial longitudinal deficiency (*p *= 0.04), thumb hypoplasia associated with radial longitudinal deficiency (*p *= 0.01), finger changes (*p *= 0.01), finger changes associated with intersegmental deficiency (*p *= 0.002), finger changes associated with radial longitudinal deficiency (*p *< 0.001), finger changes associated with thumb hypoplasia (*p *= 0.003) and having multiple congenital upper limb differences (*p *= 0.03) were associated with a significantly greater QuickDASH score, indicating more severe upper limb disability (Table S1). Multiple regression analysis showed that these collectively accounted for 17% of the variance in the QuickDASH score (*F *(7,100) = 2.817; *r*^2^ = 0.165; *p *= 0.01). Finger changes associated with radial longitudinal deficiency were the only independent predictor of a greater QuickDASH score, indicating more severe upper limb disability, when adjusting for confounding variables (*β *= 22.7; *t *= 2.3*; p *= 0.03) (Table S2).

### EQ-5D-5L index

The median EQ-5D-5L index was 0.6 (IQR 0.4 to 0.7), indicating good HRQoL. Univariate analysis confirmed that thumb hypoplasia associated with radial longitudinal deficiency (*p *= 0.03), finger changes (*p *= 0.01), finger changes associated with intersegmental deficiency (*p *< 0.001), finger changes associated with radial longitudinal deficiency (*p *= 0.001) and finger changes associated with thumb hypoplasia (*p *= 0.03) were associated with a significantly lower EQ-5D-5L index, indicating poorer HRQoL (Table S3).

### WSAS

The median WSAS was 15.5 (IQR 8.0 to 23.5), indicating considerable functional impairment. Univariate analysis confirmed that radial longitudinal deficiency (*p *= 0.01), thumb hypoplasia associated with radial longitudinal deficiency (*p *= 0.01), finger changes (*p *= 0.003), finger changes associated with intersegmental deficiency (*p *= 0.001), finger changes associated with radial longitudinal deficiency (*p *< 0.001) and finger changes associated with thumb hypoplasia (*p *= 0.02) were associated with significantly greater WSAS, indicating greater functional impairment (Table S4).

### DAS-24

The median DAS-24 was 35.5 (IQR 28.0 to 50.5), indicating poor self-perception of appearance. Univariate analysis confirmed that radial longitudinal deficiency (*p *= 0.01), thumb hypoplasia associated with radial longitudinal deficiency (*p *= 0.01), finger changes (*p *= 0.003), finger changes associated with intersegmental deficiency (*p *= 0.02), finger changes associated with radial longitudinal deficiency (*p *= 0.001), finger changes associated with thumb hypoplasia (*p *= 0.04) and having multiple congenital upper limb differences (*p *= 0.02) were associated with significantly greater DAS-24, indicating poorer self-perception of appearance (Table S5).

### NPS

The overall median NPS was −0.8 (IQR −1.4 to 0.8). Based on the NPS, 33 patients (26%) reported neuropathic pain. Univariate analysis confirmed that distal intersegmental deficiency was associated with significantly lower NPS (*p *= 0.02) (Table S6).

### Impact of surgical treatment on outcome measures

Sixty-three patients (50%) previously underwent surgical treatment of which 25 (40%) underwent bilateral surgical treatment. Forty-seven patients (75%) reported improvement in upper limb function after surgical treatment. No significant differences were found in the mean QuickDASH score, median EQ-5D-5L index, median WSAS, median DAS-24 or median NPS between patients that underwent surgical treatment and patients that underwent non-surgical treatment (Table S7).

Of note, no significant differences were found in the mean QuickDASH score, median EQ-5D-5L index, median WSAS, median DAS-24 or median NPS between patients with peromelia/amelia/phocomelia (higher chance of requiring prostheses) and patients with forearm involvement, namely radial longitudinal deficiency (Table S8).

### Impact of increasing age on HRQoL

**Figure 1.**
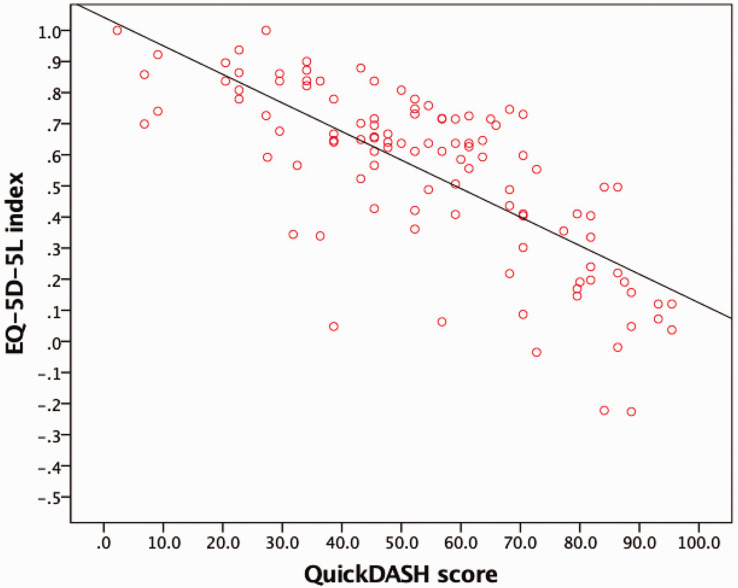
Scatter plot with line of best fit to show the correlation between the Quick Version of Disabilities of Arm, Shoulder, and Hand (QuickDASH) score and EuroQoL-5 Dimension-5 Likert (EQ-5D-5L) index.

Eighty-nine patients (70%) reported deterioration in HRQoL with increasing age, seven patients (6%) reported a gradual improvement and 23 patients (18%) reported no change. More severe upper limb disability, as quantified by the QuickDASH score, correlated strongly and significantly with HRQoL, as measured by the EQ-5D-5L index (*r *= –0.75; *p *< 0.001) (Figure 1).

### Access to healthcare

Thirty patients (24%) reported difficulty in accessing healthcare services, of which 14 (47%) reported a perceived lack of understanding of the complex needs of patients with thalidomide embryopathy from healthcare professionals as the reason for the difficulty experienced.

## Discussion

This study has reported the long-term upper limb function in patients with upper limb thalidomide embryopathy in the UK. As patients with upper limb thalidomide embryopathy enter their seventh decade of life, they report considerable upper limb disability, which correlates strongly with HRQoL. Patients also report significant functional impairment, poor self-perception of appearance and high prevalence of neuropathic pain.

The mean QuickDASH score of 54.3 (SD 22.6) suggests considerable upper limb disability. Ghassemi Jahani et al. (2014) investigated patients in Sweden and reported a mean DASH score, the full version of the QuickDASH score, of 20.5 (SD 15.6). It has been shown that DASH and QuickDASH questionnaires produce near identical scores, allowing direct comparison (Aasheim and Finsen, 2013; Gummesson et al., 2006). The upper limb disability of patients in our cohort therefore appears to be much greater than reported in Sweden. The mean age of patients in the study from Sweden was 46 years, which is considerably younger than the age of patients in our cohort (59 to 64 years). We hypothesize that the greater QuickDASH scores reported in our study may be a result of worsening upper limb disability with increasing age.

The median EQ-5D-5L index of 0.6 (IQR 0.4 to 0.7) suggests good HRQoL. Bent et al. (2007) investigated patients in the UK at the mean age of 40 years and reported a comparable median EQ-5D-3L index, an older version of the EQ-5D-5L index, of 0.6 (IQR 0.2 to 0.7). Ghassemi Jahani et al. (2016) also subsequently investigated HRQoL of patients in Sweden at the median age of 46 years and reported a mean EQ-5D-3L index of 0.6 (SD 0.1). It has been shown that the EQ-5D-3L index systematically overestimates health problems and has lower sensitivity and precision than the EQ-5D-5L index (Janssen et al., 2018; Mulhern et al., 2018). This may explain the similar scores between the studies despite the significantly greater age of patients in our cohort. The significant negative correlation between the EQ-5D-5L index and QuickDASH score observed in our study suggests poorer HRQoL with more severe upper limb disability, and a similar negative correlation between the EQ-5D-3L index and DASH score was observed in patients in Sweden (Ghassemi Jahani et al., 2016). We also found that a significant proportion of patients reported deterioration in HRQoL with increasing age. Despite surgical and non-surgical treatment, many patients experienced poorer upper limb function than healthy counterparts. We hypothesize that patients were required to compensate for poorer upper limb function throughout life by using unaffected or less severely affected limbs or body parts more frequently and for unusual tasks (Bent et al., 2007; Newbronner and Atkin, 2018). With increasing age, patients may be experiencing the accumulative impact of degenerative changes associated with overuse of these body parts: the hands, shoulders, back, hips, knees and feet are particularly affected (Bent et al., 2007; Newbronner and Atkin, 2018). This may be amplified by expected degenerative changes associated with the natural ageing process. As degenerative changes take place, patients must adjust to further declines in upper limb function and related health problems, which may explain the self-perceived deterioration in HRQoL.

It is important to also consider the concept of ‘hidden defects’. This was first proposed by Ruffing (1977) after an assessment of children with thalidomide embryopathy revealed a higher rate of specific complications, particularly block vertebrae and proximal femoral focal deficiency, which were not immediately visible on clinical examination. It was hypothesized that such hidden defects may only become apparent with ageing and may lead to a higher rate of earlier degenerative changes, particularly osteoarthritis. Furthermore, Newbronner et al. (2019) investigated patients in the UK and found that 93% reported musculoskeletal problems including pain, loss of joint movement and arthritis. This is consistent with the findings of several other studies (Bent et al., 2007; Newbronner and Atkin, 2018; Nippert et al., 2002; O’Carroll et al., 2011). We hypothesize that the emergence of hidden defects, in combination with progressive degenerative changes, may have led to our observation of greater QuickDASH scores, indicating more severe upper limb disability and an age-related deterioration in HRQoL.

Neuropathic pain was reported by 26% of patients. Several other studies also found that patients with thalidomide embryopathy reported development of neuropathic pain and neurological symptoms, such as numbness, tingling, loss of sensitivity, loss of dexterity and partial paralysis with increasing age (Newbronner and Atkin, 2018; Nicotra et al., 2016). Newbronner et al. (2019) investigated patients in the UK and found that 66% reported neurological symptoms. Jankelowitz et al. (2013) investigated a small cohort of patients in Australia and New Zealand with new-onset neurological symptoms. Clinical neurological examinations and neurophysiological investigations revealed no evidence of ongoing neuronal loss, late reactivated neuronal degeneration or generalized peripheral neuropathy. Jankelowitz et al. (2013) concluded that the new-onset neurological symptoms resulted largely from compressive neuropathies. We hypothesize that the high prevalence of neuropathic pain found in our study may also be attributed to the development of compressive neuropathies with increasing age. Compressive neuropathies may be exacerbated by patients using unaffected or less severely affected limbs or body parts more frequently and for unusual tasks to compensate for poorer upper limb function throughout life. Nicotra et al. (2016) investigated peripheral nerve function in a small cohort of patients in the UK with neurological symptoms and found that 88% had compressive neuropathies, most commonly at the wrist. It has been suggested that patients with upper limb thalidomide embryopathy are more likely to develop carpal tunnel syndrome as congenital upper limb differences and in some cases overuse of the unaffected or less severely affected upper limb may lead to narrowing of the carpal tunnel and compression of the median nerve (Jankelowitz et al., 2013).

Univariate analysis showed that finger changes associated with thumb hypoplasia were associated with a better self-perception of appearance as measured by DAS-24. This may be attributed to the less visible nature of these congenital upper limb differences and possible concealment through the adaptation of certain postures. Multiple regression analysis showed that finger changes associated with radial longitudinal deficiency were an independent predictor of greater QuickDASH scores, indicating more severe upper limb disability, despite not being considered to be as severe as other congenital upper limb differences, such as transverse and intersegmental deficiency. Further, these more severe congenital upper limb differences were not associated with poorer HRQoL or greater functional impairment, as evidenced by the univariate analysis of the EQ-5D-5L index and WSAS, respectively. In this study, we did not ask patients to describe the various types of finger changes as it was felt that a balance between using too many terminologies and language that patients are able to understand was important. Therefore, no classifications of finger changes were available. In addition, we found that more severe congenital upper limb differences were also not associated with greater prevalence of neuropathic pain, as evidenced by the univariate analysis of NPS. This may be attributed to greater resilience and acceptance among patients with more severe congenital upper limb differences that are more visible and required significant adaptations throughout life to compensate for poorer upper limb function. These patients may have come to accept their upper limb thalidomide embryopathy as an integral part of self-identity over time, which may explain these findings.

This study found that a significant proportion of patients reported improvement in upper limb function after surgical treatment. However, comparison of surgical and non-surgical treatment showed no significant differences in upper limb disability, HRQoL, functional impairment, self-perception of appearance or neuropathic pain, as evidenced by the QuickDASH score, EQ-5D-5L index, WSAS, DAS-24 and NPS, respectively. It is likely that patients with more severe congenital upper limb differences underwent surgical treatment while those with less severe differences underwent non-surgical treatment. Surgical treatment in patients with more severe congenital upper limb differences may have therefore led to an improvement in upper limb function to an extent that matched, rather than exceeded, that of patients with less severe congenital upper limb differences. At present, there are no other studies investigating the outcomes of surgical and non-surgical treatment in patients with upper limb thalidomide embryopathy. The heterogeneity of congenital upper limb differences, combined with a lack of historical record data of these procedures that were typically performed at a young age, preclude a more detailed comparison between these two groups.

A significant number of patients reported difficulties in accessing healthcare (24%). Nippert et al. (2002) investigated female patients in Germany and found that 43% reported difficulty in accessing healthcare services due to a perceived lack of understanding from healthcare professionals. Many healthcare professionals, even congenital hand specialists, are unfamiliar with upper limb thalidomide embryopathy. In addition, as these patients transitioned to adult care, most hand and upper limb surgeons are not trained in congenital hand surgery. There is a need to centralize specialist care and support of patients with upper limb thalidomide embryopathy, which will enable greater collaboration between congenital and adult hand surgery. Communication at a national level, as currently facilitated by organizations, such as the Thalidomide Trust, is vital for this purpose.

The primary limitation of this study is the non-response rate; however, a recent study suggested that this does not automatically lead to non-responder bias in studies that use the QuickDASH questionnaire (Stirling et al., 2021). Moreover, our study is strengthened by a large patient cohort, which compares favourably with previously published studies (Bent et al., 2007; Ghassemi Jahani et al., 2014, 2016; Nippert et al., 2002; O’Carroll et al., 2011). The use of validated PROMs, including those related to self-perception of appearance and activities of daily living, allowed a more holistic view of the problems faced by patients. A further limitation is the lack of demographic data, such as age and gender, which we were unable to obtain owing to requests from the Thalidomide Trust for complete anonymity to be maintained throughout. However, we were able to confirm a minimum age of 59 years as the questionnaire was sent to a cohort within the 450 patients with thalidomide embryopathy in the UK, which allowed the outcomes to be compared with studies investigating younger age groups (Bent et al., 2007; Ghassemi Jahani et al., 2014, 2016;  Newbronner et al., 2019; Nippert et al., 2002; O’Carroll et al., 2011). In addition, we collected what we considered to be more important data, such as diagnoses and previous surgical treatment.

This study has provided a comprehensive description of the long-term upper limb function in patients with upper limb thalidomide embryopathy in the UK. Patients experience considerable deterioration in upper limb function with a resultant effect on HRQoL. Furthermore, these appear to be increasing with age. We hypothesize that our findings may be explained by the emergence of hidden defects, combined with progressive deterioration associated with ageing. The care needs of patients with upper limb thalidomide embryopathy are complex and require further ongoing coordinated national efforts between specialities. It is hoped that the results of this study will highlight their needs, and can be translated to other congenital hand differences.

## Supplemental Material

sj-pdf-1-jhs-10.1177_17531934231164093 - Supplemental material for Age-related changes in patients with upper limb thalidomide embryopathy in the United Kingdom Click here for additional data file. Supplemental material, sj-pdf-1-jhs-10.1177_17531934231164093 for Age-related changes in patients with upper limb thalidomide embryopathy in the United Kingdom by Magdalena Markiewicz, Paul Stirling, Susan Brennan, Geoffrey Hooper and Wee Lam in Journal of Hand Surgery (European Volume)

sj-pdf-2-jhs-10.1177_17531934231164093 - Supplemental material for Age-related changes in patients with upper limb thalidomide embryopathy in the United Kingdom Click here for additional data file. Supplemental material, sj-pdf-2-jhs-10.1177_17531934231164093 for Age-related changes in patients with upper limb thalidomide embryopathy in the United Kingdom by Magdalena Markiewicz, Paul Stirling, Susan Brennan, Geoffrey Hooper and Wee Lam in Journal of Hand Surgery (European Volume)

sj-pdf-3-jhs-10.1177_17531934231164093 - Supplemental material for Age-related changes in patients with upper limb thalidomide embryopathy in the United Kingdom Click here for additional data file. Supplemental material, sj-pdf-3-jhs-10.1177_17531934231164093 for Age-related changes in patients with upper limb thalidomide embryopathy in the United Kingdom by Magdalena Markiewicz, Paul Stirling, Susan Brennan, Geoffrey Hooper and Wee Lam in Journal of Hand Surgery (European Volume)

sj-pdf-4-jhs-10.1177_17531934231164093 - Supplemental material for Age-related changes in patients with upper limb thalidomide embryopathy in the United Kingdom Click here for additional data file. Supplemental material, sj-pdf-4-jhs-10.1177_17531934231164093 for Age-related changes in patients with upper limb thalidomide embryopathy in the United Kingdom by Magdalena Markiewicz, Paul Stirling, Susan Brennan, Geoffrey Hooper and Wee Lam in Journal of Hand Surgery (European Volume)

sj-pdf-5-jhs-10.1177_17531934231164093 - Supplemental material for Age-related changes in patients with upper limb thalidomide embryopathy in the United Kingdom Click here for additional data file. Supplemental material, sj-pdf-5-jhs-10.1177_17531934231164093 for Age-related changes in patients with upper limb thalidomide embryopathy in the United Kingdom by Magdalena Markiewicz, Paul Stirling, Susan Brennan, Geoffrey Hooper and Wee Lam in Journal of Hand Surgery (European Volume)

sj-pdf-6-jhs-10.1177_17531934231164093 - Supplemental material for Age-related changes in patients with upper limb thalidomide embryopathy in the United Kingdom Click here for additional data file. Supplemental material, sj-pdf-6-jhs-10.1177_17531934231164093 for Age-related changes in patients with upper limb thalidomide embryopathy in the United Kingdom by Magdalena Markiewicz, Paul Stirling, Susan Brennan, Geoffrey Hooper and Wee Lam in Journal of Hand Surgery (European Volume)

sj-pdf-7-jhs-10.1177_17531934231164093 - Supplemental material for Age-related changes in patients with upper limb thalidomide embryopathy in the United Kingdom Click here for additional data file. Supplemental material, sj-pdf-7-jhs-10.1177_17531934231164093 for Age-related changes in patients with upper limb thalidomide embryopathy in the United Kingdom by Magdalena Markiewicz, Paul Stirling, Susan Brennan, Geoffrey Hooper and Wee Lam in Journal of Hand Surgery (European Volume)

sj-pdf-8-jhs-10.1177_17531934231164093 - Supplemental material for Age-related changes in patients with upper limb thalidomide embryopathy in the United Kingdom Click here for additional data file. Supplemental material, sj-pdf-8-jhs-10.1177_17531934231164093 for Age-related changes in patients with upper limb thalidomide embryopathy in the United Kingdom by Magdalena Markiewicz, Paul Stirling, Susan Brennan, Geoffrey Hooper and Wee Lam in Journal of Hand Surgery (European Volume)
