# Supplementary material for: Age-related changes in patients with upper limb thalidomide embryopathy in the United Kingdom
Source: J Hand Surg Eur Vol. 2023 Apr 6;48(8):773–80. doi: 10.1177/17531934231164093 (PMC10466990; doi:10.1177/17531934231164093)
Supplement: sj-pdf-1-jhs-10.1177_17531934231164093 - Supplemental material for Age-related changes in patients with upper limb thalidomide embryopathy in the United Kingdom [file sj-pdf-1-jhs-10.1177_17531934231164093.pdf]

Table S1. Univariate analysis for QuickDASH score.

Variable<br>(OMT classification)	QuickDASH<br>(mean, SD)	*p*-value
Unilateral amelia (I-A-1-iii-a)		
Yes	55.9 (23.0)	
No	54.3 (22.4)	0.89*
Segmental transverse deficiency (I-A-1-iii-b)		
Yes	56.8 (19.3)	
No	54.3 (22.4)	0.88*
Proximal intersegmental deficiency (I-A-1-iv-a)		
Yes	58.0 (20.3)	
No	53.9 (22.6)	0.53*
Distal intersegmental deficiency (I-A-1-iv-b)		
Yes	56.9 (22.1)	
No	53.9 (22.4)	0.61*
Proximal and distal intersegmental deficiency (I-A-1-iv-c)		
Yes	56.4 (22.0)	
No	51.4 (22.7)	0.25
Radial longitudinal deficiency (I-A-2-i)		
Yes	58.6 (22.0)	
No	49.6 (21.9)	**0.04***
Thumb hypoplasia (I-B-2-i)		
Yes	55.3 (21.2)	
No	50.1 (27.3)	0.37*
Thumb hypoplasia associated with radial longitudinal deficiency		
Yes	59.8 (21.4)	
No	49.1 (22.1)	**0.01***

Finger changes		
Yes	58.9 (20.5)	
No	46.6 (23.4)	**0.01***
Finger changes associated with intersegmental deficiency		
Yes	60.8 (19.0)	
No	47.4 (23.6)	**0.002***
Finger changes associated with radial longitudinal deficiency		
Yes	64.0 (18.0)	
No	48.2 (22.8)	**<0.001***
Finger changes associated with thumb hypoplasia		
Yes	60.4 (18.7)	
No	47.7 (24.2)	**0.003***
Multiple congenital upper limb differences		
Yes	56.3 (21.1)	
No	43.0 (26.1)	**0.03***
Surgical treatment		
Yes	55.8 (22.0)	
No	52.6 (22.7)	0.45*

OMT classification: Oberg-Manske-Tonkin classification, QuickDASH: Quick Version of the Disabilities of the Arm, Shoulder, and Hand questionnaire, SD: standard deviation.

*Independent samples *t*-test.
