# Supplementary material for: Age-related changes in patients with upper limb thalidomide embryopathy in the United Kingdom
Source: J Hand Surg Eur Vol. 2023 Apr 6;48(8):773–80. doi: 10.1177/17531934231164093 (PMC10466990; doi:10.1177/17531934231164093)
Supplement: sj-pdf-2-jhs-10.1177_17531934231164093 - Supplemental material for Age-related changes in patients with upper limb thalidomide embryopathy in the United Kingdom [file sj-pdf-2-jhs-10.1177_17531934231164093.pdf]

Table S2. Multiple regression analysis for predictors of QuickDASH score.

Variable<br>(OMT classification)	QuickDASH<br>(*beta* coefficient, 95% CI)	*p*-value
Radial longitudinal deficiency (I-A-2-i)	-21.9 (-48.7 to 4.8)	0.11
Thumb hypoplasia associated with radial longitudinal deficiency	12.3 (-11.5 to 36.1)	0.31
Finger changes	-5.2 (-24.2 to 13.9)	0.59
Finger changes associated with intersegmental deficiency	5.2 (-8.7 to 19.1)	0.46
Finger changes associated with radial longitudinal deficiency	22.7 (2.9 to 42.4)	**0.03**
Finger changes associated with thumb hypoplasia	-2.7 (-19.0 to 13.6)	0.74
Multiple congenital upper limb differences	11.1 (-4.1 to 26.4)	0.15

OMT classification: Oberg-Manske-Tonkin classification, QuickDASH: Quick Version of the Disabilities of the Arm, Shoulder, and Hand questionnaire, CI: confidence interval.
