# Supplementary material for: Age-related changes in patients with upper limb thalidomide embryopathy in the United Kingdom
Source: J Hand Surg Eur Vol. 2023 Apr 6;48(8):773–80. doi: 10.1177/17531934231164093 (PMC10466990; doi:10.1177/17531934231164093)
Supplement: sj-pdf-3-jhs-10.1177_17531934231164093 - Supplemental material for Age-related changes in patients with upper limb thalidomide embryopathy in the United Kingdom [file sj-pdf-3-jhs-10.1177_17531934231164093.pdf]

Table S3. Univariate analysis for EQ-5D-5L index.

Variable (OMT classification)	EQ-5D-5L (median, IQR)	*p*-value
Unilateral amelia (I-A-1-iii-a)		
Yes	0.6 (0.4 to 0.7)	
No	0.6 (0.4 to 0.7)	0.86*
Segmental transverse deficiency (I-A-1-iii-b)		
Yes	0.5 (0.4 to 0.6)	
No	0.6 (0.4 to 0.7)	0.68*
Proximal intersegmental deficiency (I-A-1-iv-a)		
Yes	0.5 (0.2 to 0.7)	
No	0.6 (0.4 to 0.7)	0.28*
Distal intersegmental deficiency (I-A-1-iv-b)		
Yes	0.6 (0.4 to 0.7)	
No	0.6 (0.4 to 0.7)	0.41*
Proximal and distal intersegmental deficiency (I-A-1-iv-c)		
Yes	0.6 (0.4 to 0.7)	
No	0.6 (0.4 to 0.8)	0.70*
Radial longitudinal deficiency (I-A-2-i)		
Yes	0.6 (0.3 to 0.7)	
No	0.6 (0.4 to 0.8)	0.08*
Thumb hypoplasia (I-B-2-i)		
Yes	0.6 (0.4 to 0.7)	
No	0.5 (0.4 to 0.7)	0.40*
Thumb hypoplasia associated with radial longitudinal deficiency	0.6 (0.2 to 0.7)	
Yes	0.6 (0.4 to 0.8)	

No		**0.03***
Finger changes		
Yes	0.6 (0.3 to 0.7)	
No	0.7 (0.5 to 0.8)	**0.01***
Finger changes associated with intersegmental deficiency		
Yes	0.5 (0.3 to 0.7)	
No	0.7 (0.5 to 0.8)	**<0.001***
Finger changes associated with radial longitudinal deficiency		
Yes	0.5 (0.2 to 0.6)	
No	0.7 (0.5 to 0.8)	**0.001***
Finger changes associated with thumb hypoplasia		
Yes	0.6 (0.3 to 0.7)	
No	0.6 (0.4 to 0.8)	**0.03***
Multiple congenital upper limb differences		
Yes	0.6 (0.4 to 0.7)	
No	0.6 (0.5 to 0.8)	0.52*
Surgical treatment		
Yes	0.6 (0.3 to 0.7)	
No	0.6 (0.4 to 0.7)	0.97*

OMT classification: Oberg-Manske-Tonkin classification, EQ-5D-5L: EuroQoL-5 Dimension-5 Likert index, IQR: interquartile range.

* Mann-Whitney U test.
