# Supplementary material for: Age-related changes in patients with upper limb thalidomide embryopathy in the United Kingdom
Source: J Hand Surg Eur Vol. 2023 Apr 6;48(8):773–80. doi: 10.1177/17531934231164093 (PMC10466990; doi:10.1177/17531934231164093)
Supplement: sj-pdf-4-jhs-10.1177_17531934231164093 - Supplemental material for Age-related changes in patients with upper limb thalidomide embryopathy in the United Kingdom [file sj-pdf-4-jhs-10.1177_17531934231164093.pdf]

Table S4. Univariate analysis for WSAS.

Variable (OMT classification)	WSAS (median, IQR)	*p*-value
Unilateral amelia (I-A-1-iii-a)		
Yes	16.0 (9.5 to 24.0)	
No	15.0 (8.0 to 23.0)	0.89*
Segmental transverse deficiency (I-A-1-iii-b)		
Yes	11.5 (8.0 to 15.0)	
No	16.0 (8.0 to 23.0)	0.56*
Proximal intersegmental deficiency (I-A-1-iv-a)		
Yes	17.0 (6.0 to 28.0)	
No	15.0 (8.0 to 23.0)	0.85*
Distal intersegmental deficiency (I-A-1-iv-b)		
Yes	15.0 (8.0 to 22.0)	
No	15.5 (7.0 to 23.0)	0.80*
Proximal and distal intersegmental deficiency (I-A-1-iv-c)		
Yes	16.5 (9.0 to 24.0)	
No	13.0 (6.0 to 22.0)	0.26*
Radial longitudinal deficiency (I-A-2-i)		
Yes	18.0 (11.0 to 28.0)	
No	11.5 (6.0 to 19.0)	**0.01***
Thumb hypoplasia (I-B-2-i)		
Yes	16.0 (8.0 to 23.0)	
No	15.0 (6.0 to 25.0)	0.72*
Thumb hypoplasia associated with radial longitudinal deficiency		
Yes	18.0 (13.0 to 28.0)	

No	11.5 (6.0 to 19.0)	**0.01***
Finger changes		
Yes	17.0 (11.0 to 23.0)	
No	8.0 (2.0 to 21.0)	**0.003***
Finger changes associated with intersegmental deficiency		
Yes	17.0 (13.0 to 25.0)	
No	8.0 (3.0 to 20.0)	**0.001***
Finger changes associated with radial longitudinal deficiency		
Yes	20.0 (15.0 to 30.0)	**<0.001***
No	11.0 (4.0 to 19.0)	
Finger changes associated with thumb hypoplasia		
Yes	16.0 (11.0 to 23.0)	
No	12.0 (3.0 to 21.0)	**0.02***
Multiple congenital upper limb differences		
Yes	16.0 (8.0 to 23.0)	
No	8.0 (3.0 to 20.5)	0.06*
Surgical treatment		
Yes	16.0 (8.0 to 23.5)	
No	15.0 (7.5 to 22.5)	0.51*

OMT classification: Oberg-Manske-Tonkin classification, WSAS: Work and Social Adjustment Scale, IQR: interquartile range.

* Mann-Whitney U test.
