# Supplementary material for: Age-related changes in patients with upper limb thalidomide embryopathy in the United Kingdom
Source: J Hand Surg Eur Vol. 2023 Apr 6;48(8):773–80. doi: 10.1177/17531934231164093 (PMC10466990; doi:10.1177/17531934231164093)
Supplement: sj-pdf-5-jhs-10.1177_17531934231164093 - Supplemental material for Age-related changes in patients with upper limb thalidomide embryopathy in the United Kingdom [file sj-pdf-5-jhs-10.1177_17531934231164093.pdf]

Table S5. Univariate analysis for DAS-24.

Variable (OMT classification)	DAS-24 (median, IQR)	*p*-value
Unilateral amelia (I-A-1-iii-a)		
Yes	42.0 (29.0 to 63.5)	
No	36.0 (28.5 to 51.0)	0.64*
Segmental transverse deficiency (I-A-1-iii-b)		
Yes	37.0 (25.0 to 49.0)	
No	36.0 (29.0 to 51.0)	0.77*
Proximal intersegmental deficiency (I-A-1-iv-a)		
Yes	34.0 (26.0 to 50.0)	
No	37.0 (29.0 to 51.0)	0.91*
Distal intersegmental deficiency (I-A-1-iv-b)		
Yes	39.0 (33.5 to 51.0)	
No	36.0 (28.0 to 51.0)	0.32*
Proximal and distal intersegmental deficiency (I-A-1-iv-c)		
Yes	37.0 (30.0 to 52.0)	
No	34.5 (26.0 to 50.0)	0.72*
Radial longitudinal deficiency (I-A-2-i)		
Yes	45.0 (30.0 to 57.0)	
No	34.0 (26.5 to 40.5)	**0.01***
Thumb hypoplasia (I-B-2-i)		
Yes	38.0 (29.0 to 51.5)	
No	33.0 (27.5 to 48.0)	0.42*
Thumb hypoplasia associated with radial longitudinal deficiency		
Yes	45.0 (30.0 to 57.0)	

No	34.0 (26.5 to 41.5)	**0.01***
Finger changes		
Yes	40.5 (31.0 to 55.0)	
No	33.0 (26.0 to 40.0)	**0.003***
Finger changes associated with intersegmental deficiency		
Yes	40.5 (31.0 to 55.0)	
No	33.0 (26.0 to 42.0)	**0.02***
Finger changes associated with radial longitudinal deficiency		
Yes	50.0 (33.5 to 57.0)	**0.001***
No	34.0 (26.0 to 41.5)	
Finger changes associated with thumb hypoplasia		
Yes	40.5 (31.0 to 55.0)	
No	34.0 (26.0 to 42.0)	**0.04***
Multiple congenital upper limb differences		
Yes	38.0 (30.0 to 52.0)	
No	29.0 (25.0 to 35.0)	**0.02***
Surgical treatment		
Yes	37.5 (29.0 to 51.0)	
No	36.0 (27.0 to 50.0)	0.82*

OMT classification: Oberg-Manske-Tonkin classification, DAS-24: Derriford Appearance Scale 24, IQR: interquartile range.

* Mann-Whitney U test.
