# Supplementary material for: Age-related changes in patients with upper limb thalidomide embryopathy in the United Kingdom
Source: J Hand Surg Eur Vol. 2023 Apr 6;48(8):773–80. doi: 10.1177/17531934231164093 (PMC10466990; doi:10.1177/17531934231164093)
Supplement: sj-pdf-6-jhs-10.1177_17531934231164093 - Supplemental material for Age-related changes in patients with upper limb thalidomide embryopathy in the United Kingdom [file sj-pdf-6-jhs-10.1177_17531934231164093.pdf]

Table S6. Univariate analysis for NPS.

Variable (OMT classification)	NPS (median, IQR)	*p*-value
Unilateral amelia (I-A-1-iii-a)		
Yes	-0.3 (-1.4 to 1.4)	
No	-0.9 (-1.4 to 0.7)	0.79*
Segmental transverse deficiency (I-A-1-iii-b)		
Yes	-0.3 (-1.4 to 0.7)	
No	-0.9 (-1.4 to 0.8)	0.98*
Proximal intersegmental deficiency (I-A-1-iv-a)		
Yes	-0.9 (-1.4 to 0.8)	
No	-0.9 (-1.4 to 0.7)	0.73*
Distal intersegmental deficiency (I-A-1-iv-b)		
Yes	-1.2 (-1.4 to -1.1)	
No	-0.5 (-1.4 to 0.8)	**0.02***
Proximal and distal intersegmental deficiency (I-A-1-iv-c)		
Yes	-0.4 (-1.3 to 0.9)	
No	-1.1 (-1.4 to 0.4)	0.08*
Radial longitudinal deficiency (I-A-2-i)		
Yes	-0.7 (-1.3 to 1.1)	
No	-1.1 (-1.4 to 0.2)	0.17*
Thumb hypoplasia (I-B-2-i)		
Yes	-1.0 (-1.4 to 0.8)	
No	-0.7 (-1.4 to 0.7)	0.58*
Thumb hypoplasia associated with radial longitudinal deficiency		
Yes	-0.7 (-1.3 to 1.2)	

No	-1.1 (-1.4 to 0.6)	0.21*
Finger changes		
Yes	-0.6 (-1.4 to 1.0)	
No	-1.1 (-1.4 to 0.2)	0.12*
Finger changes associated with intersegmental deficiency		
Yes	-0.6 (-1.3 to 0.9)	
No	-1.1 (-1.4 to 0.2)	0.14*
Finger changes associated with radial longitudinal deficiency	-0.6 (-1.3 to 1.3)	
Yes	-1.1 (-1.4 to 0.2)	0.12*
No		
Finger changes associated with thumb hypoplasia		
Yes	-0.9 (-1.3 to 1.0)	
No	-0.9 (-1.4 to 0.3)	0.45*
Multiple congenital upper limb differences		
Yes	-0.9 (-1.4 to 0.8)	
No	-1.2 (-1.4 to -0.1)	0.23*
Surgical treatment		
Yes	-0.5 (-1.3 to 0.8)	
No	-1.2 (-1.4 to 0.5)	0.21*

OMT classification: Oberg-Manske-Tonkin classification, NPS: Neuropathic Pain Scale, IQR: interquartile range.

* Mann-Whitney U test.
