# Supplementary material for: Age-related changes in patients with upper limb thalidomide embryopathy in the United Kingdom
Source: J Hand Surg Eur Vol. 2023 Apr 6;48(8):773–80. doi: 10.1177/17531934231164093 (PMC10466990; doi:10.1177/17531934231164093)
Supplement: sj-pdf-7-jhs-10.1177_17531934231164093 - Supplemental material for Age-related changes in patients with upper limb thalidomide embryopathy in the United Kingdom [file sj-pdf-7-jhs-10.1177_17531934231164093.pdf]

Table S7. Comparison of outcome measures between surgical and non-surgical treatment.

Outcome measure		*p*-value
QuickDASH (mean, SD)		
Surgical treatment	55.8 (22.0)	
Non-surgical treatment	52.6 (22.7)	0.45*
EQ-5D-5L (median, IQR)		
Surgical treatment	0.6 (0.2 to 0.8)	
Non-surgical treatment	0.6 (0.5 to 0.7)	0.97†
WSAS (median, IQR)		
Surgical treatment	18.0 (8.0 to 25.0)	
Non-surgical treatment	13.5 (7.0 to 22.0)	0.51†
DAS-24 (median, IQR)		
Surgical treatment	35.0 (28.0 to 50.0)	
Non-surgical treatment	36.0 (26.0 to 49.0)	0.82†
NPS (median, IQR)		
Surgical treatment	-0.4 (-1.3 to 0.8)	
Non-surgical treatment	-1.2 (-1.4 to 0.5)	0.21†

OMT classification: Oberg-Manske-Tonkin classification, QuickDASH: Quick Version of the Disabilities of the Arm, Shoulder, and Hand questionnaire, EQ-5D-5L: EuroQoL-5 Dimension-5 Likert index, WSAS: Work and Social Adjustment Scale, DAS-24: Derriford Appearance Scale 24, NPS: Neuropathic Pain Scale, SD: standard deviation, IQR: interquartile range.

*Independent samples *t*-test.

†Mann-Whitney U test.
