# Supplementary material for: Age-related changes in patients with upper limb thalidomide embryopathy in the United Kingdom
Source: J Hand Surg Eur Vol. 2023 Apr 6;48(8):773–80. doi: 10.1177/17531934231164093 (PMC10466990; doi:10.1177/17531934231164093)
Supplement: sj-pdf-8-jhs-10.1177_17531934231164093 - Supplemental material for Age-related changes in patients with upper limb thalidomide embryopathy in the United Kingdom [file sj-pdf-8-jhs-10.1177_17531934231164093.pdf]

Table S8. Comparison of outcome measures between peromelia/amelia/phocomelia and forearm involvement i.e. radial longitudinal deficiency.

Congenital upper limb difference (OMT classification)		*p*-value
QuickDASH (mean, SD)		
Transverse deficiency (I-A-1-iii) or intersegmental deficiency (I-A-1-iv)	56.0 (20.9)	
Radial longitudinal deficiency (I-A-2-i)	56.1 (22.2)	0.99*
EQ-5D-5L (median, IQR)		
Transverse deficiency (I-A-1-iii) or intersegmental deficiency (I-A-1-iv)	0.6 (0.4 to 0.7)	
Radial longitudinal deficiency (I-A-2-i)	0.6 (0.3 to 0.7)	0.97†
WSAS (median, IQR)		
Transverse deficiency (I-A-1-iii) or intersegmental deficiency (I-A-1-iv)	14.0 (8.0 to 23.0)	
Radial longitudinal deficiency (I-A-2-i)	18.0 (9.5 to 27.5)	0.52†
DAS-24 (median, IQR)		
Transverse deficiency (I-A-1-iii) or intersegmental deficiency (I-A-1-iv)	34.5 (26.0 to 44.5)	
Radial longitudinal deficiency (I-A-2-i)	40.0 (28.5 to 55.5)	0.19†
NPS (median, IQR)		
Transverse deficiency (I-A-1-iii) or intersegmental deficiency (I-A-1-iv)	-1.1 (-1.4 to 0.4)	
Radial longitudinal deficiency (I-A-2-i)	-0.7 (-1.3 to 0.9)	0.21†

OMT classification: Oberg-Manske-Tonkin classification, QuickDASH: Quick Version of the Disabilities of the Arm, Shoulder, and Hand questionnaire, EQ-5D-5L: EuroQoL-5 Dimension-5 Likert index, WSAS: Work and Social Adjustment Scale, DAS-24: Derriford Appearance Scale 24, NPS: Neuropathic Pain Scale, SD: standard deviation, IQR: interquartile range.

*Independent samples *t*-test.

[†]Mann-Whitney U test.
